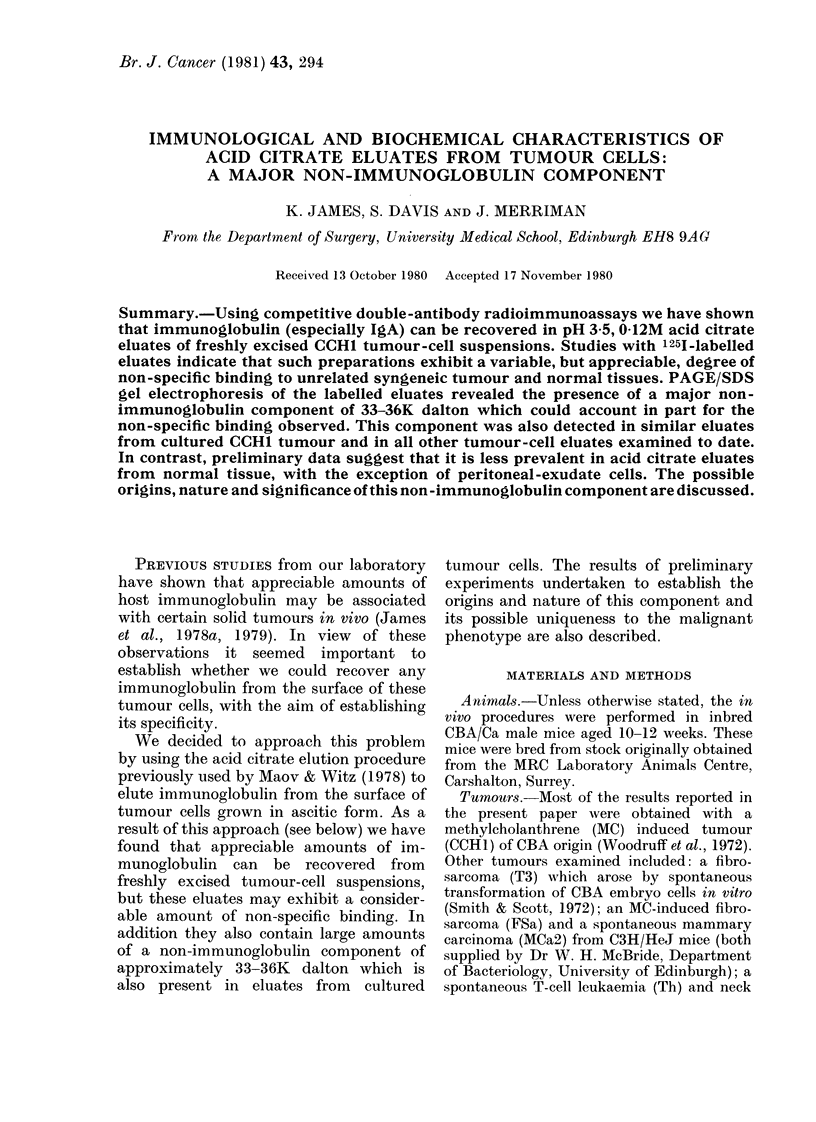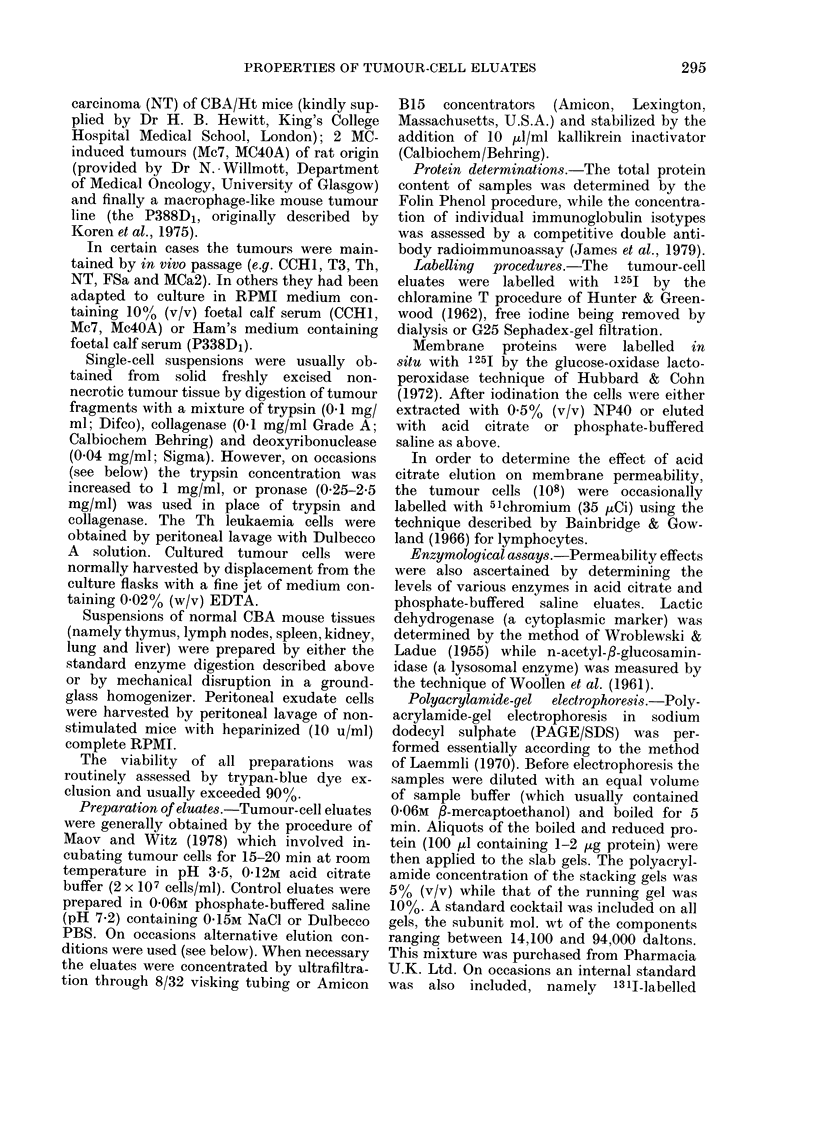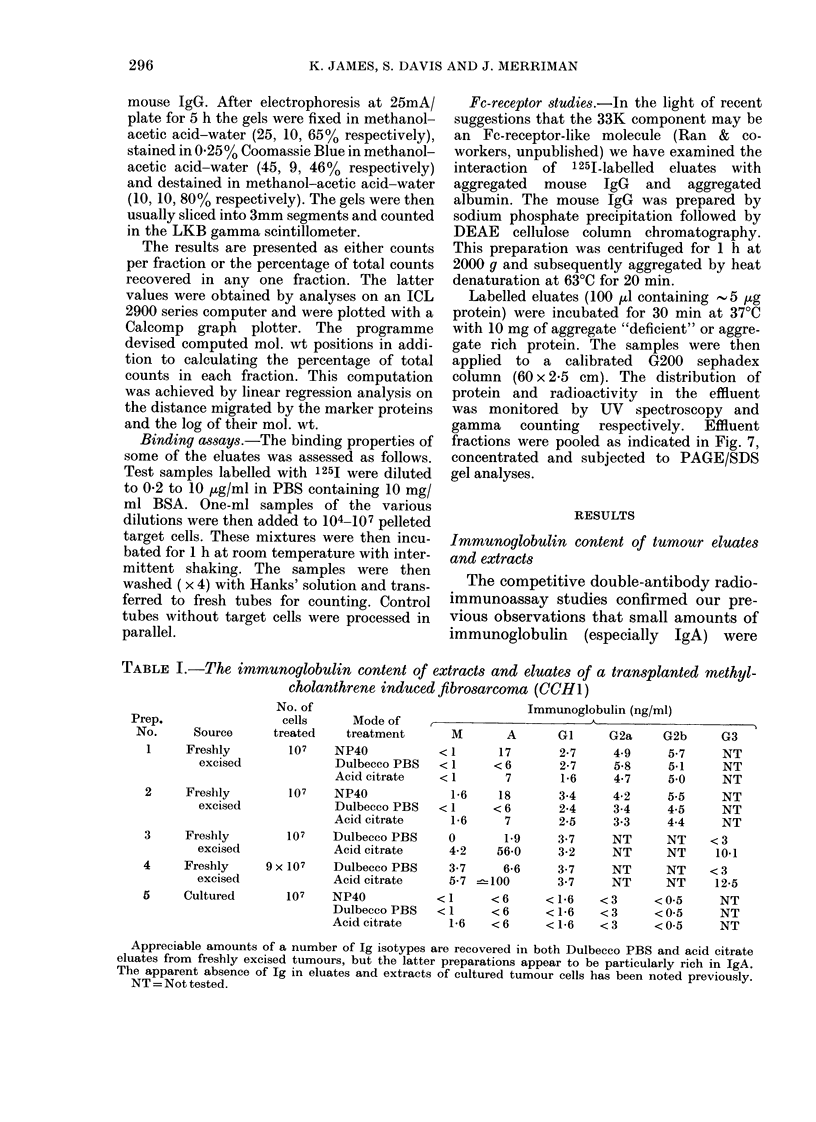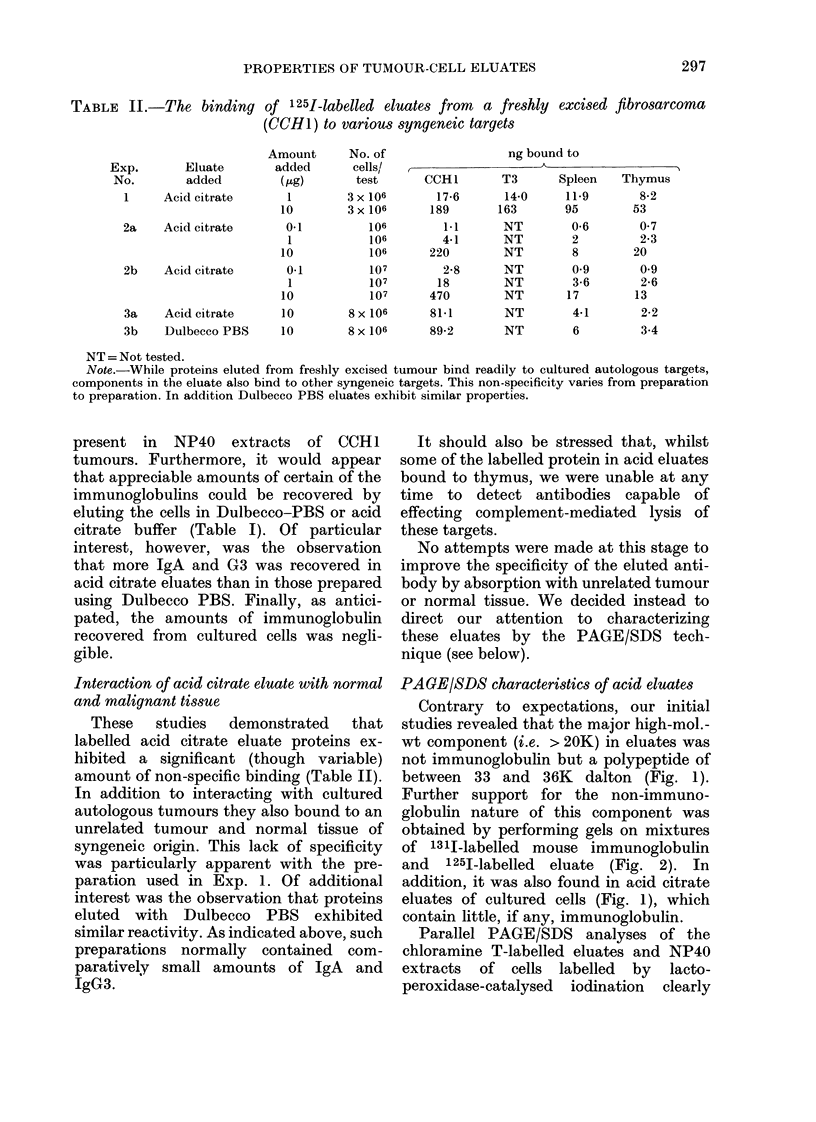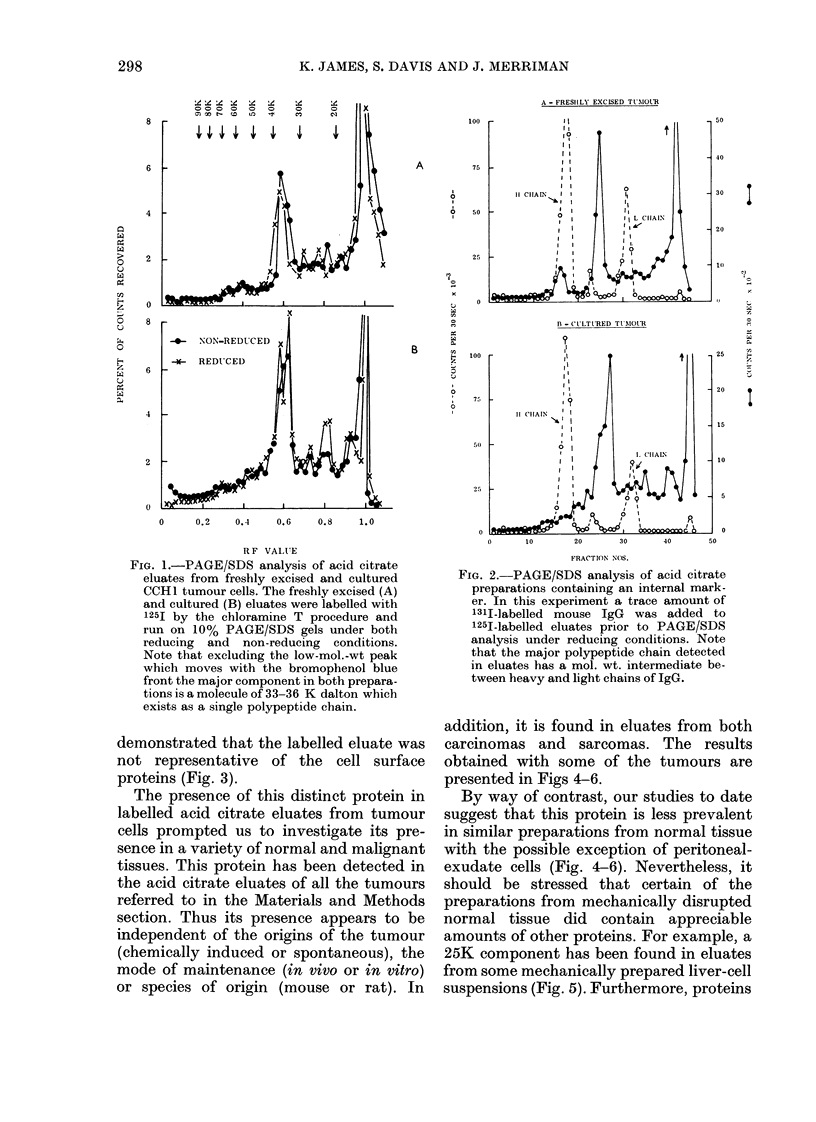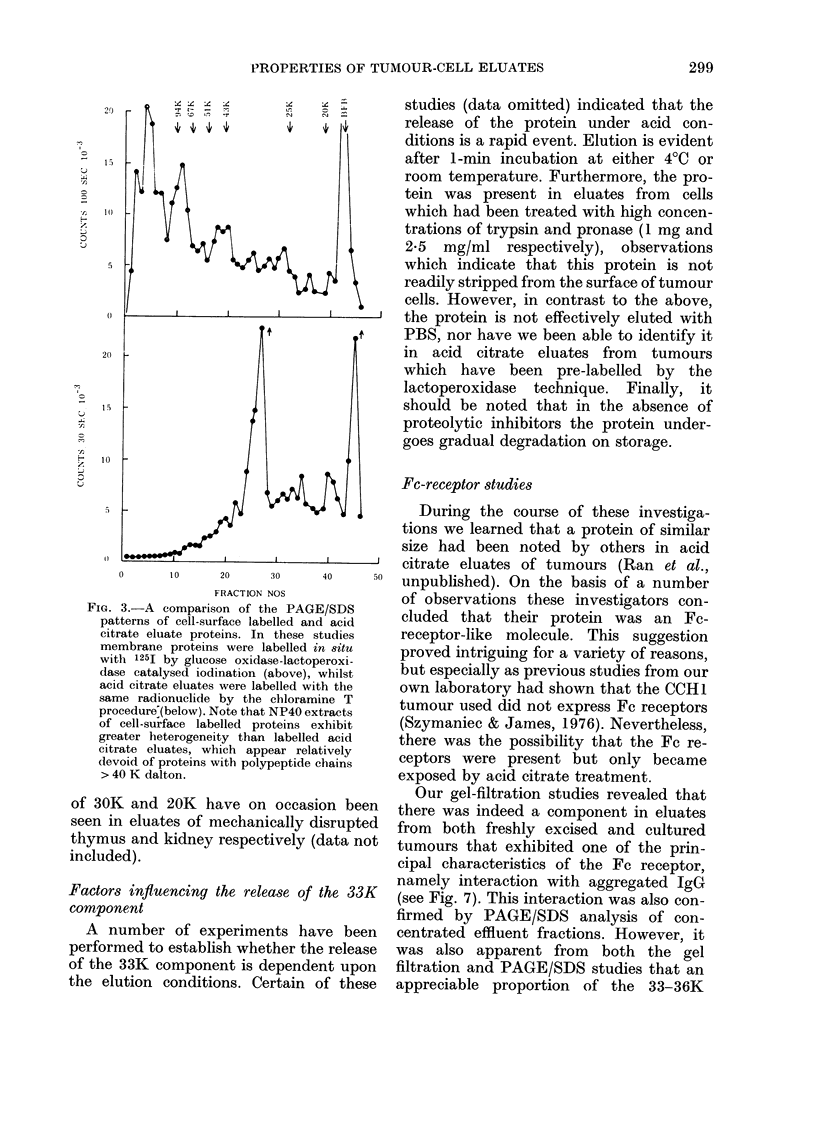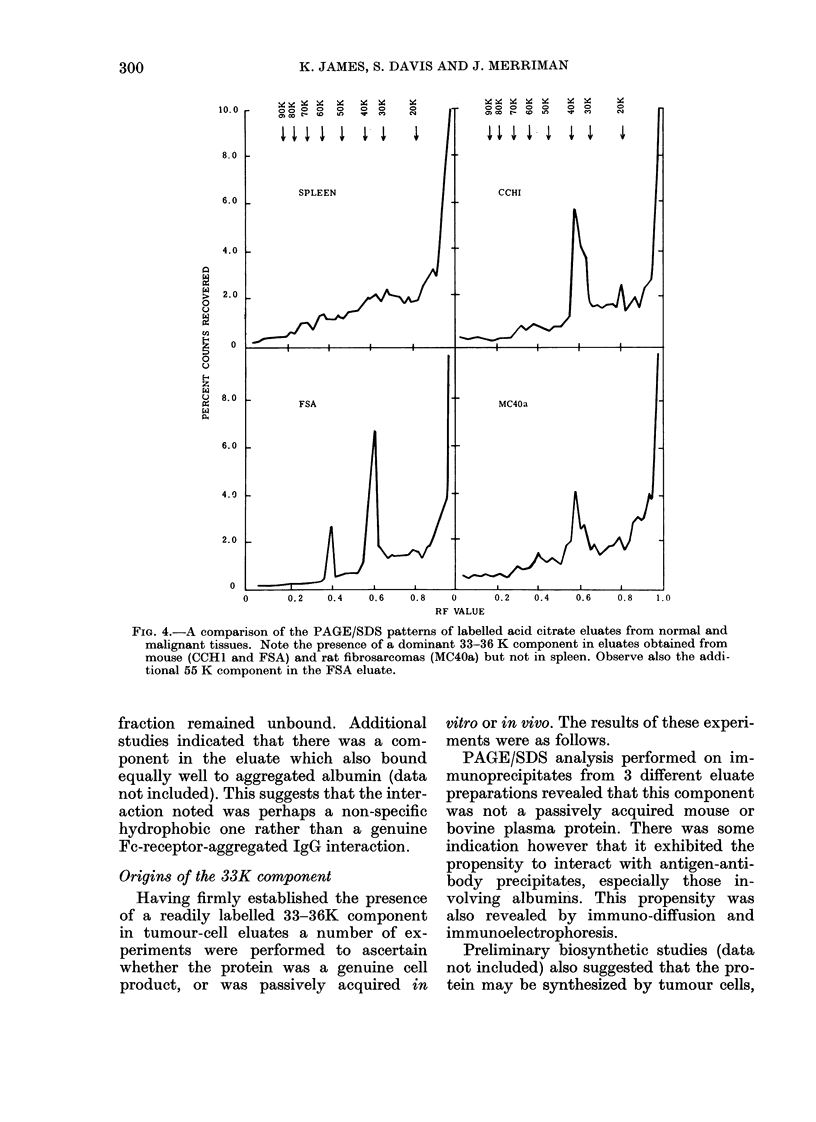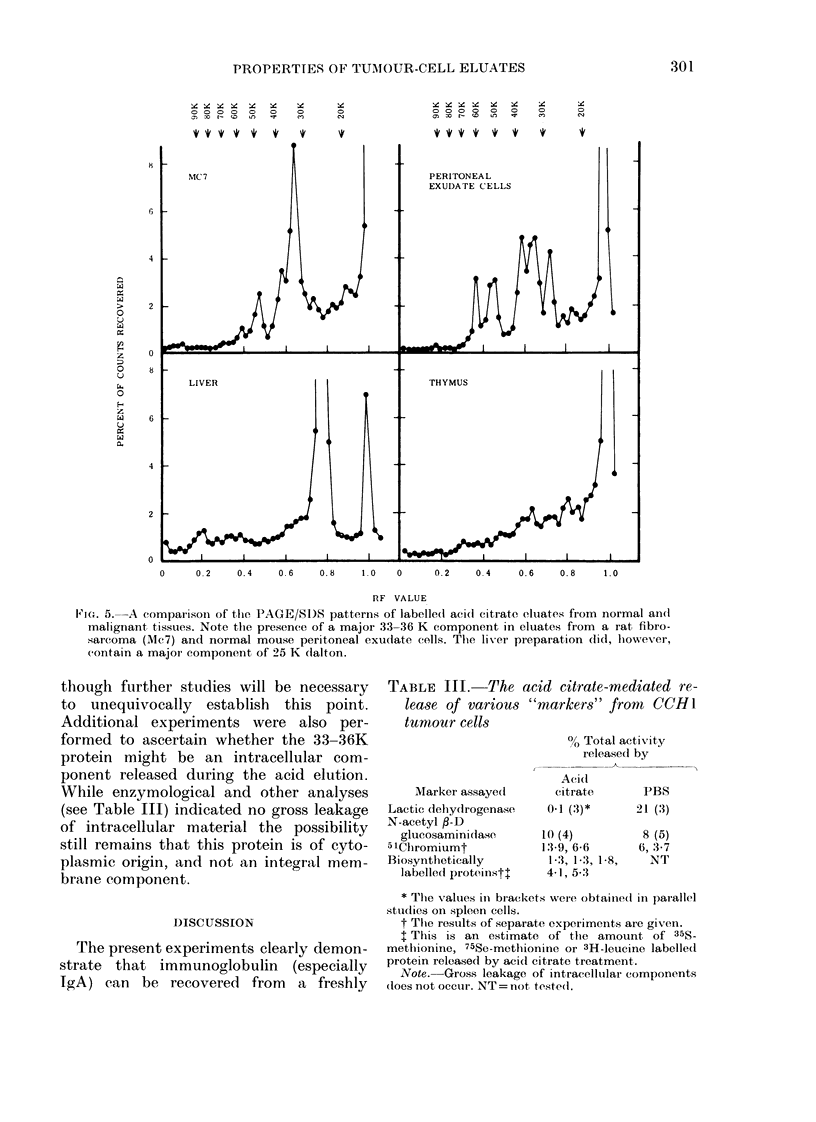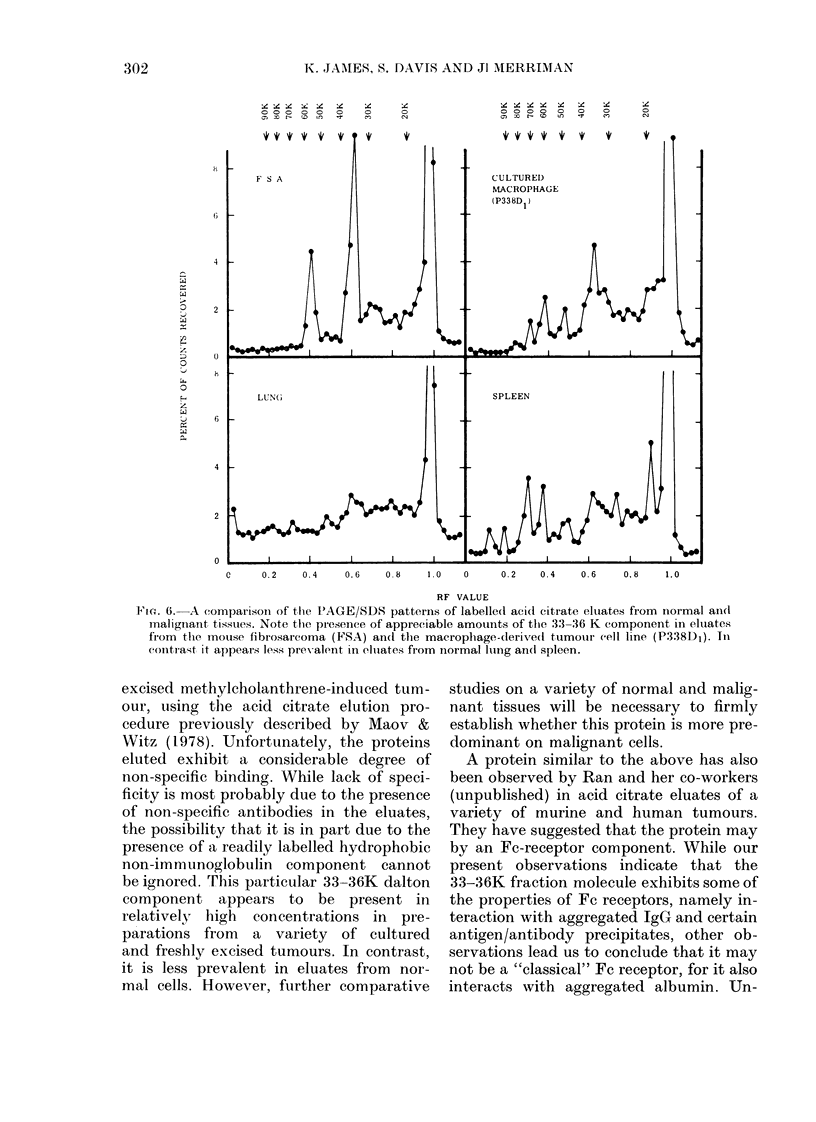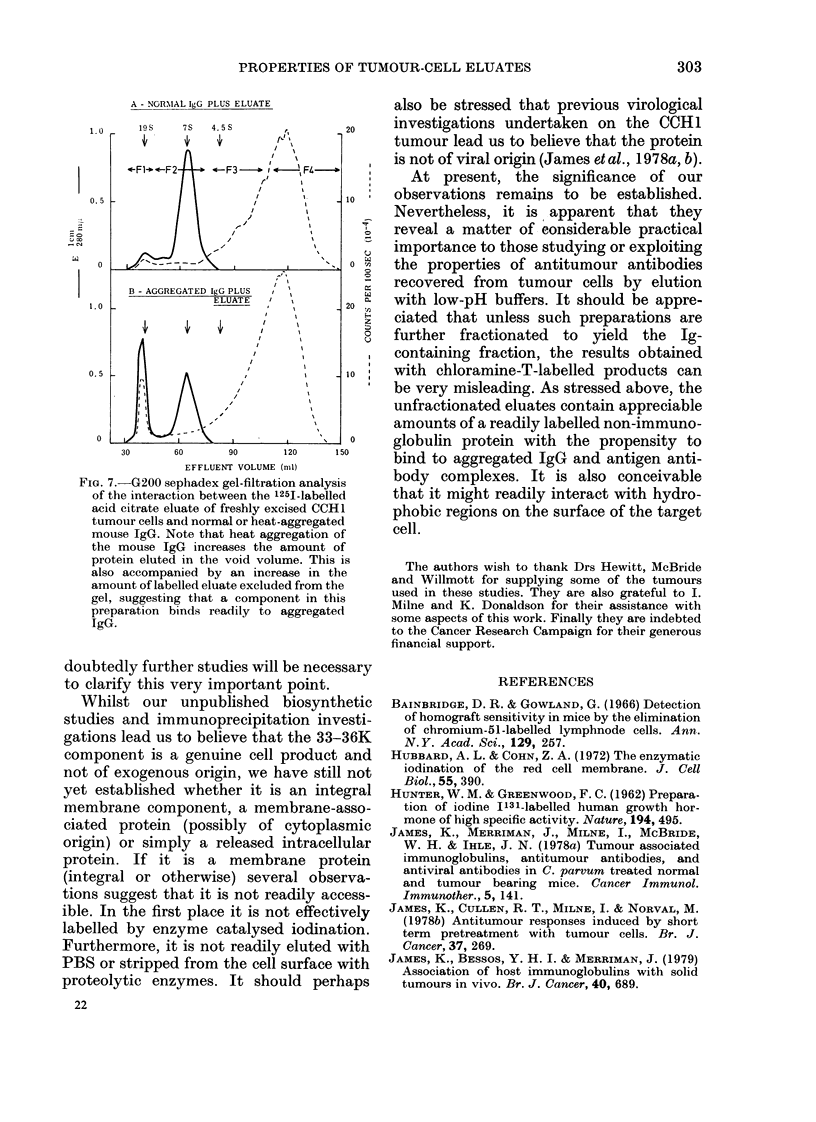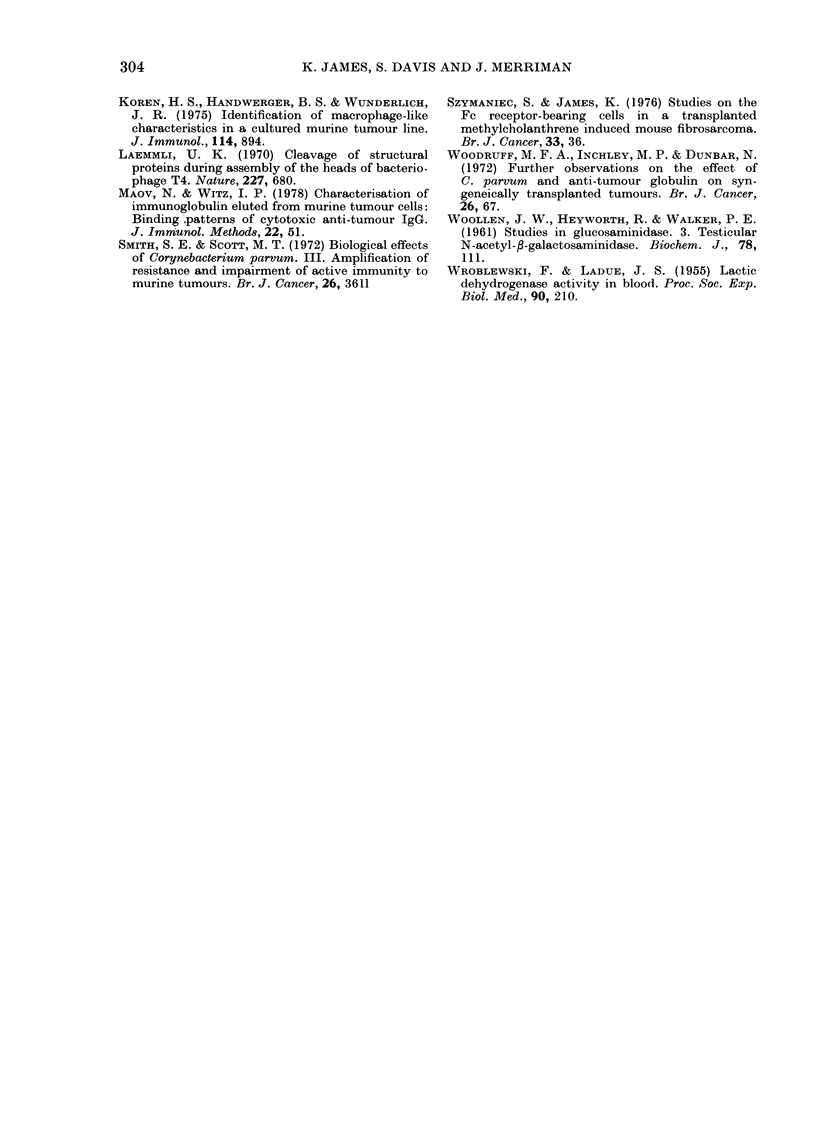# Immunological and biochemical characteristics of acid citrate eluates from tumour cells: a major non-immunoglobulin component.

**DOI:** 10.1038/bjc.1981.47

**Published:** 1981-03

**Authors:** K. James, S. Davis, J. Merriman

## Abstract

Using competitive double-antibody radioimmunoassays we have shown that immunoglobulin (especially IgA) can be recovered in pH 3.5, 0.12M acid citrate eluates of freshly excised CCH1 tumour-cell suspensions. Studies with 125I-labelled eluates indicate that such preparations exhibit a variable, but appreciable, degree of non-specific binding to unrelated syngeneic tumour and normal tissues. PAGE/SDS gel electrophoresis of the labelled eluates revealed the presence of a major non-immunoglobulin component of 33-36K dalton which could account in part for the non-specific binding observed. This component was also detected in similar eluates from cultured CCH1 tumour and in all other tumour-cell eluates examined to date. In contrast, preliminary data suggest that it is less prevalent in acid citrate eluates from normal tissue, with the exception of peritoneal-exudate cells. The possible origins, nature and significance of this non-immunoglobulin component are discussed.


*Br. J. Cancer* (1981) **43**, 294

### IMMUNOLOGICAL AND BIOCHEMICAL CHARACTERISTICS OF ACID CITRATE ELUATES FROM TUMOUR CELLS: A MAJOR NON-IMMUNOGLOBULIN COMPONENT

K. JAMES, S. DAVIS AND J. MERRIMAN

*From the Department of Surgery, University Medical School, Edinburgh EH8 9AG*

Received 13 October 1980 Accepted 17 November 1980

**Summary.—Using competitive double-antibody radioimmunoassays we have shown that immunoglobulin (especially IgA) can be recovered in pH 3·5, 0·12M acid citrate eluates of freshly excised CCH1 tumour-cell suspensions. Studies with $^{125}$I-labelled eluates indicate that such preparations exhibit a variable, but appreciable, degree of non-specific binding to unrelated syngeneic tumour and normal tissues. PAGE/SDS gel electrophoresis of the labelled eluates revealed the presence of a major non-immunoglobulin component of 33–36K dalton which could account in part for the non-specific binding observed. This component was also detected in similar eluates from cultured CCH1 tumour and in all other tumour-cell eluates examined to date. In contrast, preliminary data suggest that it is less prevalent in acid citrate eluates from normal tissue, with the exception of peritoneal-exudate cells. The possible origins, nature and significance of this non-immunoglobulin component are discussed.**

PREVIOUS STUDIES from our laboratory have shown that appreciable amounts of host immunoglobulin may be associated with certain solid tumours *in vivo* (James *et al.*, 1978*a*, 1979). In view of these observations it seemed important to establish whether we could recover any immunoglobulin from the surface of these tumour cells, with the aim of establishing its specificity.

We decided to approach this problem by using the acid citrate elution procedure previously used by Maov & Witz (1978) to elute immunoglobulin from the surface of tumour cells grown in ascitic form. As a result of this approach (see below) we have found that appreciable amounts of immunoglobulin can be recovered from freshly excised tumour-cell suspensions, but these eluates may exhibit a considerable amount of non-specific binding. In addition they also contain large amounts of a non-immunoglobulin component of approximately 33–36K dalton which is also present in eluates from cultured tumour cells. The results of preliminary experiments undertaken to establish the origins and nature of this component and its possible uniqueness to the malignant phenotype are also described.

### MATERIALS AND METHODS

*Animals*.—Unless otherwise stated, the *in vivo* procedures were performed in inbred CBA/Ca male mice aged 10–12 weeks. These mice were bred from stock originally obtained from the MRC Laboratory Animals Centre, Carshalton, Surrey.

*Tumours*.—Most of the results reported in the present paper were obtained with a methylcholanthrene (MC) induced tumour (CCH1) of CBA origin (Woodruff *et al.*, 1972). Other tumours examined included: a fibrosarcoma (T3) which arose by spontaneous transformation of CBA embryo cells *in vitro* (Smith & Scott, 1972); an MC-induced fibrosarcoma (FSa) and a spontaneous mammary carcinoma (MCa2) from C3H/HeJ mice (both supplied by Dr W. H. McBride, Department of Bacteriology, University of Edinburgh); a spontaneous T-cell leukaemia (Th) and neck carcinoma (NT) of CBA/Ht mice (kindly supplied by Dr H. B. Hewitt, King's College Hospital Medical School, London); 2 MC-induced tumours (Mc7, MC40A) of rat origin (provided by Dr N. Willmott, Department of Medical Oncology, University of Glasgow) and finally a macrophage-like mouse tumour line (the $P388D_1$, originally described by Koren *et al.*, 1975).

In certain cases the tumours were maintained by *in vivo* passage (*e.g.* CCH1, T3, Th, NT, FSa and MCa2). In others they had been adapted to culture in RPMI medium containing 10% (v/v) foetal calf serum (CCH1, Mc7, Mc40A) or Ham's medium containing foetal calf serum ($P338D_1$).

Single-cell suspensions were usually obtained from solid freshly excised non-necrotic tumour tissue by digestion of tumour fragments with a mixture of trypsin (0·1 mg/ml; Difco), collagenase (0·1 mg/ml Grade A; Calbiochem Behring) and deoxyribonuclease (0·04 mg/ml; Sigma). However, on occasions (see below) the trypsin concentration was increased to 1 mg/ml, or pronase (0·25–2·5 mg/ml) was used in place of trypsin and collagenase. The Th leukaemia cells were obtained by peritoneal lavage with Dulbecco A solution. Cultured tumour cells were normally harvested by displacement from the culture flasks with a fine jet of medium containing 0·02% (w/v) EDTA.

Suspensions of normal CBA mouse tissues (namely thymus, lymph nodes, spleen, kidney, lung and liver) were prepared by either the standard enzyme digestion described above or by mechanical disruption in a ground-glass homogenizer. Peritoneal exudate cells were harvested by peritoneal lavage of non-stimulated mice with heparinized (10 u/ml) complete RPMI.

The viability of all preparations was routinely assessed by trypan-blue dye exclusion and usually exceeded 90%.

*Preparation of eluates.*—Tumour-cell eluates were generally obtained by the procedure of Maov and Witz (1978) which involved incubating tumour cells for 15–20 min at room temperature in pH 3·5, 0·12M acid citrate buffer ($2 \times 10^7$ cells/ml). Control eluates were prepared in 0·06M phosphate-buffered saline (pH 7·2) containing 0·15M NaCl or Dulbecco PBS. On occasions alternative elution conditions were used (see below). When necessary the eluates were concentrated by ultrafiltration through 8/32 visking tubing or Amicon B15 concentrators (Amicon, Lexington, Massachusetts, U.S.A.) and stabilized by the addition of 10 $\mu$l/ml kallikrein inactivator (Calbiochem/Behring).

*Protein determinations.*—The total protein content of samples was determined by the Folin Phenol procedure, while the concentration of individual immunoglobulin isotypes was assessed by a competitive double antibody radioimmunoassay (James *et al.*, 1979).

*Labelling procedures.*—The tumour-cell eluates were labelled with $^{125}I$ by the chloramine T procedure of Hunter & Greenwood (1962), free iodine being removed by dialysis or G25 Sephadex-gel filtration.

Membrane proteins were labelled *in situ* with $^{125}I$ by the glucose-oxidase lactoperoxidase technique of Hubbard & Cohn (1972). After iodination the cells were either extracted with 0·5% (v/v) NP40 or eluted with acid citrate or phosphate-buffered saline as above.

In order to determine the effect of acid citrate elution on membrane permeability, the tumour cells ($10^8$) were occasionally labelled with $^{51}$chromium (35 $\mu$Ci) using the technique described by Bainbridge & Gowland (1966) for lymphocytes.

*Enzymological assays.*—Permeability effects were also ascertained by determining the levels of various enzymes in acid citrate and phosphate-buffered saline eluates. Lactic dehydrogenase (a cytoplasmic marker) was determined by the method of Wroblewski & Ladue (1955) while n-acetyl-$\beta$-glucosaminidase (a lysosomal enzyme) was measured by the technique of Woollen *et al.* (1961).

*Polyacrylamide-gel electrophoresis.*—Polyacrylamide-gel electrophoresis in sodium dodecyl sulphate (PAGE/SDS) was performed essentially according to the method of Laemmli (1970). Before electrophoresis the samples were diluted with an equal volume of sample buffer (which usually contained 0·06M $\beta$-mercaptoethanol) and boiled for 5 min. Aliquots of the boiled and reduced protein (100 $\mu$l containing 1–2 $\mu$g protein) were then applied to the slab gels. The polyacrylamide concentration of the stacking gels was 5% (v/v) while that of the running gel was 10%. A standard cocktail was included on all gels, the subunit mol. wt of the components ranging between 14,100 and 94,000 daltons. This mixture was purchased from Pharmacia U.K. Ltd. On occasions an internal standard was also included, namely $^{131}I$-labelled mouse IgG. After electrophoresis at 25mA/plate for 5 h the gels were fixed in methanol–acetic acid–water (25, 10, 65% respectively), stained in 0·25% Coomassie Blue in methanol–acetic acid–water (45, 9, 46% respectively) and destained in methanol–acetic acid–water (10, 10, 80% respectively). The gels were then usually sliced into 3mm segments and counted in the LKB gamma scintillometer.

The results are presented as either counts per fraction or the percentage of total counts recovered in any one fraction. The latter values were obtained by analyses on an ICL 2900 series computer and were plotted with a Calcomp graph plotter. The programme devised computed mol. wt positions in addition to calculating the percentage of total counts in each fraction. This computation was achieved by linear regression analysis on the distance migrated by the marker proteins and the log of their mol. wt.

*Binding assays.*—The binding properties of some of the eluates was assessed as follows. Test samples labelled with $^{125}I$ were diluted to 0·2 to 10 μg/ml in PBS containing 10 mg/ml BSA. One-ml samples of the various dilutions were then added to $10^4$–$10^7$ pelleted target cells. These mixtures were then incubated for 1 h at room temperature with intermittent shaking. The samples were then washed (×4) with Hanks' solution and transferred to fresh tubes for counting. Control tubes without target cells were processed in parallel.

*Fc-receptor studies.*—In the light of recent suggestions that the 33K component may be an Fc-receptor-like molecule (Ran & coworkers, unpublished) we have examined the interaction of $^{125}I$-labelled eluates with aggregated mouse IgG and aggregated albumin. The mouse IgG was prepared by sodium phosphate precipitation followed by DEAE cellulose column chromatography. This preparation was centrifuged for 1 h at 2000 *g* and subsequently aggregated by heat denaturation at 63°C for 20 min.

Labelled eluates (100 μl containing ~5 μg protein) were incubated for 30 min at 37°C with 10 mg of aggregate "deficient" or aggregate rich protein. The samples were then applied to a calibrated G200 sephadex column (60×2·5 cm). The distribution of protein and radioactivity in the effluent was monitored by UV spectroscopy and gamma counting respectively. Effluent fractions were pooled as indicated in Fig. 7, concentrated and subjected to PAGE/SDS gel analyses.

### RESULTS

### *Immunoglobulin content of tumour eluates and extracts*

The competitive double-antibody radioimmunoassay studies confirmed our previous observations that small amounts of immunoglobulin (especially IgA) were

TABLE I.—*The immunoglobulin content of extracts and eluates of a transplanted methylcholanthrene induced fibrosarcoma (CCH1)*

| Prep. No. | Source | No. of cells treated | Mode of treatment | Immunoglobulin (ng/ml) M | A | G1 | G2a | G2b | G3 |
|---|---|---|---|---|---|---|---|---|---|
| 1 | Freshly excised | $10^7$ | NP40 | <1 | 17 | 2·7 | 4·9 | 5·7 | NT |
| | | | Dulbecco PBS | <1 | <6 | 2·7 | 5·8 | 5·1 | NT |
| | | | Acid citrate | <1 | 7 | 1·6 | 4·7 | 5·0 | NT |
| 2 | Freshly excised | $10^7$ | NP40 | 1·6 | 18 | 3·4 | 4·2 | 5·5 | NT |
| | | | Dulbecco PBS | <1 | <6 | 2·4 | 3·4 | 4·5 | NT |
| | | | Acid citrate | 1·6 | 7 | 2·5 | 3·3 | 4·4 | NT |
| 3 | Freshly excised | $10^7$ | Dulbecco PBS | 0 | 1·9 | 3·7 | NT | NT | <3 |
| | | | Acid citrate | 4·2 | 56·0 | 3·2 | NT | NT | 10·1 |
| 4 | Freshly excised | $9\times10^7$ | Dulbecco PBS | 3·7 | 6·6 | 3·7 | NT | NT | <3 |
| | | | Acid citrate | 5·7 | ≏100 | 3·7 | NT | NT | 12·5 |
| 5 | Cultured | $10^7$ | NP40 | <1 | <6 | <1·6 | <3 | <0·5 | NT |
| | | | Dulbecco PBS | <1 | <6 | <1·6 | <3 | <0·5 | NT |
| | | | Acid citrate | 1·6 | <6 | <1·6 | <3 | <0·5 | NT |

Appreciable amounts of a number of Ig isotypes are recovered in both Dulbecco PBS and acid citrate eluates from freshly excised tumours, but the latter preparations appear to be particularly rich in IgA. The apparent absence of Ig in eluates and extracts of cultured tumour cells has been noted previously. NT = Not tested.

TABLE II.—*The binding of $^{125}I$-labelled eluates from a freshly excised fibrosarcoma (CCH1) to various syngeneic targets*

| Exp. No. | Eluate added | Amount added (μg) | No. of cells/ test | ng bound to CCH1 | T3 | Spleen | Thymus |
|---|---|---|---|---|---|---|---|
| 1 | Acid citrate | 1 | $3\times10^6$ | 17·6 | 14·0 | 11·9 | 8·2 |
| | | 10 | $3\times10^6$ | 189 | 163 | 95 | 53 |
| 2a | Acid citrate | 0·1 | $10^6$ | 1·1 | NT | 0·6 | 0·7 |
| | | 1 | $10^6$ | 4·1 | NT | 2 | 2·3 |
| | | 10 | $10^6$ | 220 | NT | 8 | 20 |
| 2b | Acid citrate | 0·1 | $10^7$ | 2·8 | NT | 0·9 | 0·9 |
| | | 1 | $10^7$ | 18 | NT | 3·6 | 2·6 |
| | | 10 | $10^7$ | 470 | NT | 17 | 13 |
| 3a | Acid citrate | 10 | $8\times10^6$ | 81·1 | NT | 4·1 | 2·2 |
| 3b | Dulbecco PBS | 10 | $8\times10^6$ | 89·2 | NT | 6 | 3·4 |

NT = Not tested.

*Note.*—While proteins eluted from freshly excised tumour bind readily to cultured autologous targets, components in the eluate also bind to other syngeneic targets. This non-specificity varies from preparation to preparation. In addition Dulbecco PBS eluates exhibit similar properties.

present in NP40 extracts of CCH1 tumours. Furthermore, it would appear that appreciable amounts of certain of the immunoglobulins could be recovered by eluting the cells in Dulbecco–PBS or acid citrate buffer (Table I). Of particular interest, however, was the observation that more IgA and G3 was recovered in acid citrate eluates than in those prepared using Dulbecco PBS. Finally, as anticipated, the amounts of immunoglobulin recovered from cultured cells was negligible.

*Interaction of acid citrate eluate with normal and malignant tissue*

These studies demonstrated that labelled acid citrate eluate proteins exhibited a significant (though variable) amount of non-specific binding (Table II). In addition to interacting with cultured autologous tumours they also bound to an unrelated tumour and normal tissue of syngeneic origin. This lack of specificity was particularly apparent with the preparation used in Exp. 1. Of additional interest was the observation that proteins eluted with Dulbecco PBS exhibited similar reactivity. As indicated above, such preparations normally contained comparatively small amounts of IgA and IgG3.

It should also be stressed that, whilst some of the labelled protein in acid eluates bound to thymus, we were unable at any time to detect antibodies capable of effecting complement-mediated lysis of these targets.

No attempts were made at this stage to improve the specificity of the eluted antibody by absorption with unrelated tumour or normal tissue. We decided instead to direct our attention to characterizing these eluates by the PAGE/SDS technique (see below).

*PAGE/SDS characteristics of acid eluates*

Contrary to expectations, our initial studies revealed that the major high-mol.-wt component (*i.e.* > 20K) in eluates was not immunoglobulin but a polypeptide of between 33 and 36K dalton (Fig. 1). Further support for the non-immunoglobulin nature of this component was obtained by performing gels on mixtures of $^{131}I$-labelled mouse immunoglobulin and $^{125}I$-labelled eluate (Fig. 2). In addition, it was also found in acid citrate eluates of cultured cells (Fig. 1), which contain little, if any, immunoglobulin.

Parallel PAGE/SDS analyses of the chloramine T-labelled eluates and NP40 extracts of cells labelled by lactoperoxidase-catalysed iodination clearly

FIG. 1.—PAGE/SDS analysis of acid citrate eluates from freshly excised and cultured CCH1 tumour cells. The freshly excised (A) and cultured (B) eluates were labelled with $^{125}$I by the chloramine T procedure and run on 10% PAGE/SDS gels under both reducing and non-reducing conditions. Note that excluding the low-mol.-wt peak which moves with the bromophenol blue front the major component in both preparations is a molecule of 33–36 K dalton which exists as a single polypeptide chain.

FIG. 2.—PAGE/SDS analysis of acid citrate preparations containing an internal marker. In this experiment a trace amount of $^{131}$I-labelled mouse IgG was added to $^{125}$I-labelled eluates prior to PAGE/SDS analysis under reducing conditions. Note that the major polypeptide chain detected in eluates has a mol. wt. intermediate between heavy and light chains of IgG.

demonstrated that the labelled eluate was not representative of the cell surface proteins (Fig. 3).

The presence of this distinct protein in labelled acid citrate eluates from tumour cells prompted us to investigate its presence in a variety of normal and malignant tissues. This protein has been detected in the acid citrate eluates of all the tumours referred to in the Materials and Methods section. Thus its presence appears to be independent of the origins of the tumour (chemically induced or spontaneous), the mode of maintenance (*in vivo* or *in vitro*) or species of origin (mouse or rat). In addition, it is found in eluates from both carcinomas and sarcomas. The results obtained with some of the tumours are presented in Figs 4–6.

By way of contrast, our studies to date suggest that this protein is less prevalent in similar preparations from normal tissue with the possible exception of peritoneal-exudate cells (Fig. 4–6). Nevertheless, it should be stressed that certain of the preparations from mechanically disrupted normal tissue did contain appreciable amounts of other proteins. For example, a 25K component has been found in eluates from some mechanically prepared liver-cell suspensions (Fig. 5). Furthermore, proteins

FIG. 3.—A comparison of the PAGE/SDS patterns of cell-surface labelled and acid citrate eluate proteins. In these studies membrane proteins were labelled *in situ* with $^{125}I$ by glucose oxidase-lactoperoxidase catalysed iodination (above), whilst acid citrate eluates were labelled with the same radionuclide by the chloramine T procedure (below). Note that NP40 extracts of cell-surface labelled proteins exhibit greater heterogeneity than labelled acid citrate eluates, which appear relatively devoid of proteins with polypeptide chains > 40 K dalton.

of 30K and 20K have on occasion been seen in eluates of mechanically disrupted thymus and kidney respectively (data not included).

*Factors influencing the release of the 33K component*

A number of experiments have been performed to establish whether the release of the 33K component is dependent upon the elution conditions. Certain of these studies (data omitted) indicated that the release of the protein under acid conditions is a rapid event. Elution is evident after 1-min incubation at either 4°C or room temperature. Furthermore, the protein was present in eluates from cells which had been treated with high concentrations of trypsin and pronase (1 mg and 2·5 mg/ml respectively), observations which indicate that this protein is not readily stripped from the surface of tumour cells. However, in contrast to the above, the protein is not effectively eluted with PBS, nor have we been able to identify it in acid citrate eluates from tumours which have been pre-labelled by the lactoperoxidase technique. Finally, it should be noted that in the absence of proteolytic inhibitors the protein undergoes gradual degradation on storage.

*Fc-receptor studies*

During the course of these investigations we learned that a protein of similar size had been noted by others in acid citrate eluates of tumours (Ran *et al.*, unpublished). On the basis of a number of observations these investigators concluded that their protein was an Fc-receptor-like molecule. This suggestion proved intriguing for a variety of reasons, but especially as previous studies from our own laboratory had shown that the CCH1 tumour used did not express Fc receptors (Szymaniec & James, 1976). Nevertheless, there was the possibility that the Fc receptors were present but only became exposed by acid citrate treatment.

Our gel-filtration studies revealed that there was indeed a component in eluates from both freshly excised and cultured tumours that exhibited one of the principal characteristics of the Fc receptor, namely interaction with aggregated IgG (see Fig. 7). This interaction was also confirmed by PAGE/SDS analysis of concentrated effluent fractions. However, it was also apparent from both the gel filtration and PAGE/SDS studies that an appreciable proportion of the 33–36K

FIG. 4.—A comparison of the PAGE/SDS patterns of labelled acid citrate eluates from normal and malignant tissues. Note the presence of a dominant 33–36 K component in eluates obtained from mouse (CCH1 and FSA) and rat fibrosarcomas (MC40a) but not in spleen. Observe also the additional 55 K component in the FSA eluate.

fraction remained unbound. Additional studies indicated that there was a component in the eluate which also bound equally well to aggregated albumin (data not included). This suggests that the interaction noted was perhaps a non-specific hydrophobic one rather than a genuine Fc-receptor-aggregated IgG interaction.

*Origins of the 33K component*

Having firmly established the presence of a readily labelled 33–36K component in tumour-cell eluates a number of experiments were performed to ascertain whether the protein was a genuine cell product, or was passively acquired *in vitro* or *in vivo*. The results of these experiments were as follows.

PAGE/SDS analysis performed on immunoprecipitates from 3 different eluate preparations revealed that this component was not a passively acquired mouse or bovine plasma protein. There was some indication however that it exhibited the propensity to interact with antigen-antibody precipitates, especially those involving albumins. This propensity was also revealed by immuno-diffusion and immunoelectrophoresis.

Preliminary biosynthetic studies (data not included) also suggested that the protein may be synthesized by tumour cells,

FIG. 5.—A comparison of the PAGE/SDS patterns of labelled acid citrate eluates from normal and malignant tissues. Note the presence of a major 33–36 K component in eluates from a rat fibrosarcoma (Mc7) and normal mouse peritoneal exudate cells. The liver preparation did, however, contain a major component of 25 K dalton.

though further studies will be necessary to unequivocally establish this point. Additional experiments were also performed to ascertain whether the 33–36K protein might be an intracellular component released during the acid elution. While enzymological and other analyses (see Table III) indicated no gross leakage of intracellular material the possibility still remains that this protein is of cytoplasmic origin, and not an integral membrane component.

TABLE III.—*The acid citrate-mediated release of various "markers" from CCH*1 *tumour cells*

| Marker assayed | % Total activity released by: Acid citrate | PBS |
|---|---|---|
| Lactic dehydrogenase | 0·1 (3)* | 21 (3) |
| N-acetyl β-D glucosaminidase | 10 (4) | 8 (5) |
| $^{51}$Chromium† | 13·9, 6·6 | 6, 3·7 |
| Biosynthetically labelled proteins†‡ | 1·3, 1·3, 1·8, 4·1, 5·3 | NT |

* The values in brackets were obtained in parallel studies on spleen cells.

† The results of separate experiments are given.

‡ This is an estimate of the amount of $^{35}$S-methionine, $^{75}$Se-methionine or $^{3}$H-leucine labelled protein released by acid citrate treatment.

*Note*.—Gross leakage of intracellular components does not occur. NT = not tested.

### DISCUSSION

The present experiments clearly demonstrate that immunoglobulin (especially IgA) can be recovered from a freshly

FIG. 6.—A comparison of the PAGE/SDS patterns of labelled acid citrate eluates from normal and malignant tissues. Note the presence of appreciable amounts of the 33–36 K component in eluates from the mouse fibrosarcoma (FSA) and the macrophage-derived tumour cell line ($P338D_1$). In contrast it appears less prevalent in eluates from normal lung and spleen.

excised methylcholanthrene-induced tumour, using the acid citrate elution procedure previously described by Maov & Witz (1978). Unfortunately, the proteins eluted exhibit a considerable degree of non-specific binding. While lack of specificity is most probably due to the presence of non-specific antibodies in the eluates, the possibility that it is in part due to the presence of a readily labelled hydrophobic non-immunoglobulin component cannot be ignored. This particular 33–36K dalton component appears to be present in relatively high concentrations in preparations from a variety of cultured and freshly excised tumours. In contrast, it is less prevalent in eluates from normal cells. However, further comparative studies on a variety of normal and malignant tissues will be necessary to firmly establish whether this protein is more predominant on malignant cells.

A protein similar to the above has also been observed by Ran and her co-workers (unpublished) in acid citrate eluates of a variety of murine and human tumours. They have suggested that the protein may by an Fc-receptor component. While our present observations indicate that the 33–36K fraction molecule exhibits some of the properties of Fc receptors, namely interaction with aggregated IgG and certain antigen/antibody precipitates, other observations lead us to conclude that it may not be a "classical" Fc receptor, for it also interacts with aggregated albumin. Un-

FIG. 7.—G200 sephadex gel-filtration analysis of the interaction between the $^{125}$I-labelled acid citrate eluate of freshly excised CCH1 tumour cells and normal or heat-aggregated mouse IgG. Note that heat aggregation of the mouse IgG increases the amount of protein eluted in the void volume. This is also accompanied by an increase in the amount of labelled eluate excluded from the gel, suggesting that a component in this preparation binds readily to aggregated IgG.

doubtedly further studies will be necessary to clarify this very important point.

Whilst our unpublished biosynthetic studies and immunoprecipitation investigations lead us to believe that the 33–36K component is a genuine cell product and not of exogenous origin, we have still not yet established whether it is an integral membrane component, a membrane-associated protein (possibly of cytoplasmic origin) or simply a released intracellular protein. If it is a membrane protein (integral or otherwise) several observations suggest that it is not readily accessible. In the first place it is not effectively labelled by enzyme catalysed iodination. Furthermore, it is not readily eluted with PBS or stripped from the cell surface with proteolytic enzymes. It should perhaps also be stressed that previous virological investigations undertaken on the CCH1 tumour lead us to believe that the protein is not of viral origin (James *et al.*, 1978*a, b*).

At present, the significance of our observations remains to be established. Nevertheless, it is apparent that they reveal a matter of considerable practical importance to those studying or exploiting the properties of antitumour antibodies recovered from tumour cells by elution with low-pH buffers. It should be appreciated that unless such preparations are further fractionated to yield the Ig-containing fraction, the results obtained with chloramine-T-labelled products can be very misleading. As stressed above, the unfractionated eluates contain appreciable amounts of a readily labelled non-immunoglobulin protein with the propensity to bind to aggregated IgG and antigen antibody complexes. It is also conceivable that it might readily interact with hydrophobic regions on the surface of the target cell.

The authors wish to thank Drs Hewitt, McBride and Willmott for supplying some of the tumours used in these studies. They are also grateful to I. Milne and K. Donaldson for their assistance with some aspects of this work. Finally they are indebted to the Cancer Research Campaign for their generous financial support.

### REFERENCES

BAINBRIDGE, D. R. & GOWLAND, G. (1966) Detection of homograft sensitivity in mice by the elimination of chromium-51-labelled lymphnode cells. *Ann. N.Y. Acad. Sci.*, **129**, 257.

HUBBARD, A. L. & COHN, Z. A. (1972) The enzymatic iodination of the red cell membrane. *J. Cell Biol.*, **55**, 390.

HUNTER, W. M. & GREENWOOD, F. C. (1962) Preparation of iodine $I^{131}$-labelled human growth hormone of high specific activity. *Nature*, **194**, 495.

JAMES, K., MERRIMAN, J., MILNE, I., MCBRIDE, W. H. & IHLE, J. N. (1978*a*) Tumour associated immunoglobulins, antitumour antibodies, and antiviral antibodies in *C. parvum* treated normal and tumour bearing mice. *Cancer Immunol. Immunother.*, **5**, 141.

JAMES, K., CULLEN, R. T., MILNE, I. & NORVAL, M. (1978*b*) Antitumour responses induced by short term pretreatment with tumour cells. *Br. J. Cancer*, **37**, 269.

JAMES, K., BESSOS, Y. H. I. & MERRIMAN, J. (1979) Association of host immunoglobulins with solid tumours in vivo. *Br. J. Cancer*, **40**, 689.

KOREN, H. S., HANDWERGER, B. S. & WUNDERLICH, J. R. (1975) Identification of macrophage-like characteristics in a cultured murine tumour line. *J. Immunol.*, **114**, 894.

LAEMMLI, U. K. (1970) Cleavage of structural proteins during assembly of the heads of bacteriophage T4. *Nature*, **227**, 680.

MAOV, N. & WITZ, I. P. (1978) Characterisation of immunoglobulin eluted from murine tumour cells: Binding patterns of cytotoxic anti-tumour IgG. *J. Immunol. Methods*, **22**, 51.

SMITH, S. E. & SCOTT, M. T. (1972) Biological effects of *Corynebacterium parvum*. III. Amplification of resistance and impairment of active immunity to murine tumours. *Br. J. Cancer*, **26**, 3611

SZYMANIEC, S. & JAMES, K. (1976) Studies on the Fc receptor-bearing cells in a transplanted methylcholanthrene induced mouse fibrosarcoma. *Br. J. Cancer*, **33**, 36.

WOODRUFF, M. F. A., INCHLEY, M. P. & DUNBAR, N. (1972) Further observations on the effect of *C. parvum* and anti-tumour globulin on syngeneically transplanted tumours. *Br. J. Cancer*, **26**, 67.

WOOLLEN, J. W., HEYWORTH, R. & WALKER, P. E. (1961) Studies in glucosaminidase. 3. Testicular N-acetyl-β-galactosaminidase. *Biochem. J.*, **78**, 111.

WROBLEWSKI, F. & LADUE, J. S. (1955) Lactic dehydrogenase activity in blood. *Proc. Soc. Exp. Biol. Med.*, **90**, 210.